# 

**Published:** 1891-03-07

**Authors:** 


					fVlllDUIfSlE 3^ ww S lOIHULSAOQ
Km INFANTS
INFANTS %1 *? ML and INYALIDS.
No. 232. Vol; IX.] Saturday,
March 7th, 1891.
[One Penny/

				

